# Differential Susceptibility to Infectious Respiratory Diseases between Males and Females Linked to Sex-Specific Innate Immune Inflammatory Response

**DOI:** 10.3389/fimmu.2017.01806

**Published:** 2017-12-13

**Authors:** Mustapha Chamekh, Maud Deny, Marta Romano, Nicolas Lefèvre, Francis Corazza, Jean Duchateau, Georges Casimir

**Affiliations:** ^1^Inflammation Unit, Laboratory of Pediatric Research, Faculty of Medicine, Queen Fabiola University Children’s Hospital, Université Libre de Bruxelles, Brussels, Belgium; ^2^Service of Immunology, Scientific Institute for Public Health (WIV-ISP), Brussels, Belgium; ^3^Laboratory of Translational Research, Faculty of Medicine, Université Libre de Bruxelles, Brussels, Belgium; ^4^Department of Pulmonology, Allergology and Cystic Fibrosis, Queen Fabiola University Children’s Hospital, Brussels, Belgium

**Keywords:** infectious respiratory diseases, sex bias, inflammatory response, X-linked innate genes, miRNAs

## Abstract

It is widely acknowledged that males and females exhibit contrasting degrees of susceptibility to infectious and non-infectious inflammatory diseases. This is particularly observed in respiratory diseases where human males are more likely to be affected by infection-induced acute inflammations compared to females. The type and magnitude of the innate immune inflammatory response play a cardinal role in this sex bias. Animal models mimicking human respiratory diseases have been used to address the biological factors that could explain the distinct outcomes. In this review, we focus on our current knowledge about experimental studies investigating sex-specific differences in infection-induced respiratory diseases and we provide an update on the most important innate immune mechanisms that could explain sex bias of the inflammatory response. We also discuss whether conclusions drawn from animal studies could be relevant to human.

## Introduction

Respiratory infections caused by a wide range of microbial pathogens are the most common diseases affecting humans worldwide leading to high rates of hospitalization in childhood and elderly. Human susceptibility to pneumoniae is clearly affected by sex as demonstrated by numerous clinical studies (1–4). We have previously reviewed this clinical aspect, so it will not be explored in further detail here (4). It is becoming clear that the severity of inflammatory symptoms vary between males and females according to the pathogen species and the type and magnitude of the inflammatory response triggered along the infection. Although there is an accumulating evidence for sex-specific differences in susceptibility to number of infectious respiratory diseases, the mechanisms at work are still scarce. Studies on murine infection models which are free of many confounders may enhance our understanding of the underlying biological factors. There are few animal studies in general and those affecting lung tissues in particular where sex impact on infection course was taken into account. In this review, we will provide an overview of infectious models used to study the sex effect on the disease severity and we will summarize the most important innate immune factors that could account for sex differences in innate immune inflammatory response. We will discuss their relevance to human and we will highlight the gaps and perspectives regarding future research developments.

## Distinct Outcomes of Infectious Pneumoniae in Males and Females and Relation with Inflammatory Response

Differences in susceptibility to lung infectious diseases between males and females have been shown in mouse models using varying clinically relevant microorganisms. It has been shown that intranasal inoculation of mice with the bacteria *Streptococcus pneumoniae*, the main etiological agent of pneumonia, results in a higher susceptibility of males compared to females (5). This was correlated with an early sharp increase of the multiplication of the bacteria in the lung, although the overall bacterial burden in males and females was similar. Males exhibited higher inflammatory response with massive infiltration of neutrophils within pulmonary tissues and increased levels of cytokines and chemokines such as IL-17A, CXCL1, and CXCL2. Likewise, in an infectious model of the pathogenic bacteria *Klebsiella pneumoniae*, male mice were also more susceptible than females (6). The same trend in favor of females was observed in murine infection by *Mycoplasma pulmonis* using mice of different genetic background (7). Higher mortality was observed in males compared to females and this was associated with dense inflammatory cell infiltrates within pulmonary alveoli in males. However, there was no significant difference in the number of *Mycoplasma* recovered from the lung or in the serum anti-*Mycoplasma* IgM response between males and females. Studies in murine models of mycobacterial infections have also shown sex bias with males being more severely affected than females [reviewed in Ref. (8)].

There are, however, some studies reporting that the advantage of females in controlling the infection and inflammation does not apply in certain infectious models. For instance, female mice infected with influenza virus had greatest impairment in the lung physiological function and produced higher levels of interferon (IFN)-γ and MCP-1 in bronchoalveolar lavage (BAL) fluids when compared to male mice, yet, no difference could be detected in the viral titer within the lung or in the BAL inflammatory cell recruitment (9). Female mice were also found to be more affected by a challenge with the opportunistic pathogen *Pseudomonas aeruginosa* than male mice, in the sense that a higher amount of bacteria and an increased level of expression of TNF-α and CXCL1 were observed within female lung tissues (10). However, these differences in bacteria burden and inflammatory response did not impact the survival rates between males and females and no sex difference could be seen regarding the numbers of polymorphonuclear cells, macrophages, and lymphocytes in the BAL fluids.

Collectively, these animal studies demonstrated a sexual dimorphism in the severity of pneumonia caused by various respiratory pathogens. In most cases, the severity of symptoms was found to correlate with a strong innate immune response triggered at the early phase of infection, but not to the overall number of invading microorganisms. Worth noting, the sex bias has been observed in different mouse strains, although most of the studies have used the C57BL/6 background (5–7, 9, 10). The clearance of invasive pathogens relies on the inflammatory and protective immune response whose magnitude should be tightly controlled, yet in males, the induced inflammatory response seems to be excessive and deleterious. These studies are consistent with the finding that human males are a risk factor for a number of infectious diseases. For example, in human tuberculosis, the gender bias is clearly established with a male:female ratio of 1.6:1 reported for 2015 (WHO Global tuberculosis report 2016). As illustrated in a number of infectious respiratory diseases, men are more likely to develop severe airway inflammatory symptoms consisting mainly on polymorphonuclear neutrophil accumulation and elevated expression of cytokines and chemokines including IL-8, TNF-α, and IL-1β (1–4, 11–13). Females generally had more favorable outcome, particularly when virulent pathogens are endowed with high inflammatory potential as is frequently the case of bacterial respiratory pathogens. Whether males or females are more affected relies not only on the pathogen species and its inflammatory potential but also on specific features of the host including age and genetic background.

## Potential Role of Toll-Like Receptors (TLRs) and X-Linked Innate Immune Genes in Sex Bias of the Inflammatory Response

The immune mechanisms underlying sex differences in susceptibility to infectious inflammatory diseases have not been fully delineated. The cause of this bias is probably multifactorial and includes sexual hormones and genetic background. We will not explore in the current review the role of sex steroids as this has been largely reviewed elsewhere (8, 14–16). Sex differences in many infectious and non-infectious inflammatory diseases are observed in all age groups including premature infants; therefore, one can consider that sexual hormones cannot fully explain this sex bias (4). We will focus on evidence highlighting the potent effect of TLRs and X chromosome-linked genes explored in different inflammatory settings.

### Potential Role of TLRs

The control of the pathogen dissemination relies on an immediate inflammatory response triggered by the innate immune system. The initiation of the innate immunity is achieved through the recognition of molecular structures broadly shared among various microorganisms (pathogen-associated molecular patterns) by specific receptors (pathogen-recognition receptors, PRRs) expressed on innate cells. TLRs are examples of PRRs allowing microbial sensing by immune cells. Upon ligation with specific pathogen motifs, i.e., lipopolysaccharides (LPS) for TLR4 and lipopeptides for TLR2, TLR transmits signals through adaptor proteins like MyD88 which recruit different kinases including IL-1 receptor-associated kinases and, therefore, lead to NF-κB and MAP kinases activation and induction of inflammatory cytokines and chemokines (17). Differences in the level of TLR4 and TLR2 expression between males and females have been suggested as a mechanism that could partially explain sex differences in inflammatory response. For instance, it has been shown that macrophages from male mice expressed higher levels of TLR4 compared to females following LPS endotoxic shock, hence contributing to excessive and deleterious inflammatory cytokine production in males (18, 19). In coxsackievirus infection model, increased expression of TLR4 has been also observed on splenic monocytes, dendritic cells, and CD3^+^, CD4^+^ lymphocytes from males compared to females, suggesting its potential implication in the disease severity (20). In this infectious model, resistance of female mice to coxsackievirus was found to correlate with higher TLR2 expression (20). However, there are some divergent reports showing either no difference in TLR4 expression on macrophages between males and females (21) or increased expression of TLR4 in female mice (22). The latter study argued that despite the higher expression of TLR4 in females, the magnitude of the inflammatory response is likely counterbalanced by resident immunomodulatory CD4^+^ T-lymphocytes that are more prevalent in resting tissues of females than males (22). Whether the level of expression of TLR4 or TLR2 in males and females could have a significant impact in the sex bias of the inflammatory response needs further investigations. On the other hand, studies on endosomal TLR9 and TLR7 have shown their potent implication in sex differences in innate immunity. TLR9 sense non-methylated CpG-containing microbial genomic DNA and induce the production of type I IFNs that are crucial in promoting a protective immunity. A low level of expression of TLR9 was suggested as a factor contributing to the higher susceptibility of female mice to cytomegalovirus infection (23). Since TLR7/8 are among innate genes located on the X chromosome, evidence of their potential contribution will be summarized in the next paragraph.

### Potential Role of X Chromosome-Linked Innate Immune Genes and MicroRNAs

A number of innate immune genes are located on the X chromosome both in human and mice and this may have significant consequences on their expression in males and females. Because males have one X chromosome, and females have two, one of the X chromosomes is randomly inactivated in females to assure an equal gene expression with males. Consequently, females have mosaic cells expressing two X-linked gene alleles, which is considered as a great advantage to cope with genetic diseases associated with recessive mutations occurring on the X chromosome (24). Among X-linked genes, about 15% escape inactivation and 10% have variable degree of inactivation (25). This may lead to an overexpression of some X-linked genes in females and if innate immune genes are affected by this silencing escape, it can result in a differential innate immune response between males and females. Females have, therefore, an advantageous genetic diversity that may explain their improved survival from number of infectious inflammatory diseases.

#### Innate Immune Genes

The chromosome X contains TLR7/8 encoding genes that are crucial in sensing viral single-strand RNAs (ssRNAs) and inducing a protective type I IFN response. It should be noted that TLR7/8 can also recognize ssRNAs from phagosomal Gram-positive bacteria, as shown in conventional dendritic cells (26). The induction of TLR7/8-dependent type I IFN in the early phase of infection is crucial for the host defense against invasive pathogens by promoting a protective inflammatory response (27). Differences in the overall expression of TLR7/8 on innate cells between males and females have not been reported. A study performed in a humanized mouse model has shown that TLR7 ligation resulted in a higher frequency of IFN-α- and TNF-α-producing plasmacytoid dendritic cells (pDCs) in females compared to males (28). In human, there are very few studies that sought to pinpoint how TLRs can influence the innate immune response in men and women. Plasmacytoid DCs from women were shown to exhibit a higher TLR7-mediated IFN-α production when compared to pDCs from men (29). This was attributed to a stronger activation rather than overexpression of TLR7 in females. Considering the important role of pDCs in inducing a protective immunity to viral pathogens, this may have a significant effect on the disease progression in men and women (30). Increased activation of TLR7-dependent immune response was shown, however, to be associated with chronic inflammations in females like in systemic lupus erythematosus (31). On the other hand, different studies have shown that TLR8 gene polymorphism has sex-specific effects in some infectious diseases, like in human tuberculosis where men are more severely affected than women (32, 33).

The cascade of TLR signaling and NF-κB activation involve the recruitment of kinases including IL-1 receptor-associated kinase-1 (IRAK-1) (34). IRAK-1 is the most studied gene regarding the sex bias of the inflammatory response. Interestingly, IRAK-1 is located on the X chromosome and is considered among genes that escape the X inactivation process, which may favor an enhanced NF-κB-dependent gene transcription in females (25). The chromosome X inactivation escape combined with the chromosome X mosaicism may favor an effective innate immune inflammatory response in females leading to a better outcome of infection-induced acute inflammations. The impact of IRAK-1 mosaicism on sex bias of the inflammatory response has been investigated in mice and human. In a mouse model of inflammatory colitis, IRAK-1 was shown to have a sex-specific role in the evolution of the disease (35). Notably, mosaicism of IRAK-1 expression in mice results in an immune cell deficiency leading to an improved sepsis outcome, as in IRAK-1-deficient mice (36). In human, a variant IRAK-1 haplotype with persistent increase of kinase activity was associated with a strong NF-κB activation and a severe inflammation (37–39). Recently, a study on cord blood cells revealed higher expression of IRAK-1 in female neonates compared to males and this was suggested as an immune advantage for females in infection-induced inflammatory diseases (40). Whether the overexpression of IRAK-1 is accompanied by an increased kinase activity was not shown in this study. Provided this is true, how this finding is an immune advantage for female neonates remains to be reconciled with previous reports arguing that the upregulation of IRAK-1 is associated with a severe inflammation while its downregulation improved sepsis (36, 37). This discrepancy may be related to the particular status of the neonatal innate immune cells compared to adults (41).

#### MicroRNAs

miRNAs are small non-coding RNAs of about 22 nucleotides. They have emerged in the last decade as key negative regulators of genes implicated in divers biological processes, through binding to and repressing translation of complementary messenger RNA (42, 43). They are transcribed either in intergenic or intronic regions of the genome. While intronic miRNAs are coregulated with their host genes, intergenic miRNAs have independent transcription units (44–46). The role of miRNAs in modulating the immune response is now well recognized (47–49). The aberrant expression of miRNAs has been associated with a broad range of inflammatory diseases (50–54). Noteworthy, X chromosome is highly enriched in genes encoding miRNAs (10% of total miRNAs) compared to Y chromosome, with an order of density twofold higher than on autosomes both in mice and humans (28). Around 50% of identified X-linked miRNAs are shared between human and mice. For instance, number of reports described the crucial role of X-linked miR223 and miR106a in the differentiation of neutrophils and monocytes, the key cell players of the innate immunity at early stages of infection. Studies on miR-223-deficient mice revealed higher numbers of granulocytes and hypermature neutrophils, indicating that miR-223 negatively regulates granulocyte generation and maturation (55, 56). Furthermore, miR223 KO mice were shown to develop severe inflammatory symptoms upon LPS challenge and increased oxidative burst of neutrophils upon infection with *Candida albicans* (55). A higher susceptibility to *Mycobacterium tuberculosis* infection associated with excessive neutrophil accumulation within the lung and tissue damages was also observed in miR223 KO versus wild-type mice (57). In this study, miR223 was shown to target CXCL2, a chemokine involved in neutrophil recruitment. In mice aerosolized with LPS, a time-dependent increase of miR223 was correlated with a reduced expression of TNF-α, CXCL1, and CXCL2 (58). In human, miR223 was shown to be involved in the granulopoiesis (59) and patients suffering from sepsis exhibited a marked decrease of miR223 expression (60). All these data point toward the importance of X-linked miR223 in the control of the immune inflammatory response by regulating neutrophil recruitment. On the other hand, Fontana et al. found miR106a to be functionally involved in the negative regulation of monocyte differentiation and maturation (61). It is, therefore, reasonable to speculate that a potent silencing escape affecting X-linked miRNAs like miR223 and miR106a could have a direct impact on the control of the fate lineage determination of hematopoietic progenitors, and consequently, could significantly impact the magnitude of the innate inflammatory response in males and females. Whether X-linked miRNAs are subjected to silencing escape and exhibit a differential expression profile between males and females under inflammatory settings are interesting ways to explore.

## Concluding Remarks and Perspectives

**Figure 1:**
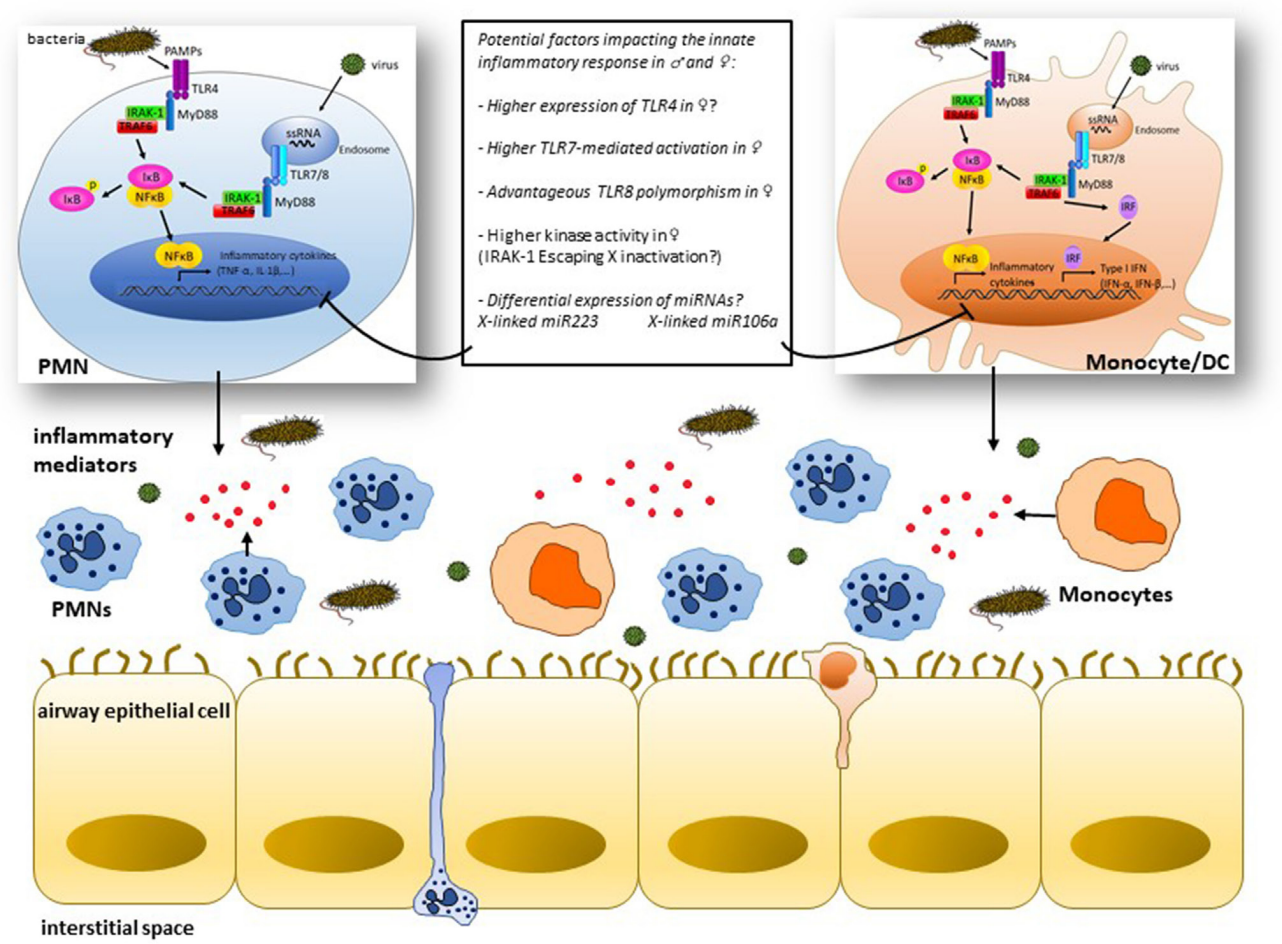
Simplified view of some mechanisms of cell type-specific signaling downstream of toll-like receptors (TLRs) potentially implicated in the sex bias of infection-induced airway inflammation: pathogen-associated molecular patterns (PAMPs) like lipopolysaccharides and single-strand RNA (ssRNA) interact with innate cells, i.e., polymorphonuclear (PMNs) cells and monocytes/dendritic cells through surface expressed TLR4 or endosomal TLR7/8, respectively, leading to the recruitment of adaptor molecules like MyD88 and activation of IL-1 receptor-associated kinase-1 (IRAK-1) among other kinases that culminate through TNF receptor-associated factors (TRAFs) with the activation of the transcription factors NF-κB and interferon regulatory factors (IRFs) and subsequent production of the inflammatory mediators (TNF-α, IL-1β, etc.) and type I interferon (IFN) gene products. Differences in the expression and/or activation of one of the cascade signaling partners between males and females may result in distinct inflammatory responses. Innate genes lying on the X chromosome, e.g., IRAK-1, likely influence the magnitude of the inflammatory response in females through a potent escape from X inactivation process. X chromosome-linked miR223 and miR106a are potentially key miRNAs that may contribute to the contrasting outcome of the inflammatory response in males and females by regulating the innate immune signaling in PMNs and monocytes, respectively.

Although caution should be made when extrapolating animal studies to human, it is now agreed that the differential outcome frequently observed in infectious respiratory diseases between males and females is likely a consequence of excessive and damaging inflammatory response rather than microbial burden within host tissues. The evidence currently available suggest the potential implication of TLRs and X-linked innate immune genes in this sex bias. However, these studies remain incomplete and sometimes conflicting. More investigations are needed to study not only the actual role of TLR signaling in the sex bias of the innate immune response but also the possible implication of other intracellular PRRs sensing nucleic acids including NOD-like and AIM2-like receptors. As schematically illustrated in Figure 1, some concepts are emerging, yet, the underlying mechanisms remain to be unraveled. Despite the considerable progress in miRNA biology and their role in the regulation of the immune and inflammatory response, their impact on sex differences of inflammatory diseases remains to be elucidated. Sex-biased expression of miRNAs could control directly the differential expression of genes contributing to sexually dimorphic inflammatory response. This stresses the need for systematic studies that aim at determining whether X-linked innate immune genes and miRNAs could be differentially expressed in male and female patients suffering from inflammatory diseases. Also, longitudinal studies are required to contrast the dynamic process of molecular signatures with the evolution of the disease outcome. Such investigations would be a pivotal step toward defining sex-specific biomarkers that are clinically relevant and whose biological function could be explored in experimental models of infection and inflammation.

## Author Contributions

MC reviewed the literature and drafted the manuscript. MD reviewed the literature and drafted the figure. MR reviewed the literature, reviewed and edited the manuscript. NL, FC, and JD reviewed the manuscript. GC reviewed and edited the manuscript.

## Conflict of Interest Statement

The authors declare that the research was conducted in the absence of any commercial or financial relationships that could be construed as a potential conflict of interest.
